# Analysis of Prion Strains by PrP^Sc^ Profiling in Sporadic Creutzfeldt–Jakob Disease

**DOI:** 10.1371/journal.pmed.0030014

**Published:** 2005-12-20

**Authors:** Gaby Schoch, Harald Seeger, Julien Bogousslavsky, Markus Tolnay, Robert Charles Janzer, Adriano Aguzzi, Markus Glatzel

**Affiliations:** **1**Institute of Neuropathology, University Hospital of Zürich, Zürich, Switzerland; **2**Department of Neurology and Division of Neuropathology, Centre Hospitalier Universitaire Vaudois, Lausanne, Switzerland; **3**Institute of Pathology, Department of Neuropathology, University Hospital Basel, Basel, Switzerland; National Institutes of HealthUnited States of America

## Abstract

**Background:**

Prion diseases are a group of invariably fatal neurodegenerative disorders affecting humans and a wide range of mammals. An essential part of the infectious agent, termed the prion, is composed of an abnormal isoform (PrP^Sc^) of a host-encoded normal cellular protein (PrP^C^). The conversion of PrP^C^ to PrP^Sc^ is thought to play a crucial role in the development of prion diseases and leads to PrP^Sc^ deposition, mainly in the central nervous system. Sporadic Creutzfeldt–Jakob disease (sCJD), the most common form of human prion disease, presents with a marked clinical heterogeneity. This diversity is accompanied by a molecular signature which can be defined by histological, biochemical, and genetic means. The molecular classification of sCJD is an important tool to aid in the understanding of underlying disease mechanisms and the development of therapy protocols. Comparability of classifications is hampered by disparity of applied methods and inter-observer variability.

**Methods and Findings:**

To overcome these difficulties, we developed a new quantification protocol for PrP^Sc^ by using internal standards on each Western blot, which allows for generation and direct comparison of individual PrP^Sc^ profiles. By studying PrP^Sc^ profiles and PrP^Sc^ type expression within nine defined central nervous system areas of 50 patients with sCJD, we were able to show distinct PrP^Sc^ distribution patterns in diverse subtypes of sCJD. Furthermore, we were able to demonstrate the co-existence of more than one PrP^Sc^ type in individuals with sCJD in about 20% of all patients and in more than 50% of patients heterozygous for a polymorphism on codon 129 of the gene encoding the prion protein *(PRNP)*.

**Conclusion:**

PrP^Sc^ profiling represents a valuable tool for the molecular classification of human prion diseases and has important implications for their diagnosis by brain biopsy. Our results show that the co-existence of more than one PrP^Sc^ type might be influenced by genetic and brain region–specific determinants. These findings provide valuable insights into the generation of distinct PrP^Sc^ types.

## Abstract 2

Internal standards allow meaningful comparison of PrP^Sc^ profiles from different origins. Applying the new method to 50 postmortem samples from patients with sporadic CJD reveals substantial differences in molecular pathology.

## Introduction

Transmissible spongiform encephalopathies or prion diseases are neurodegenerative disorders characterized by posttranslational conversion and cerebral accumulation of a pathological isoform (PrP^Sc^) of a host-encoded membrane-associated glycoprotein (cellular prion protein, PrP^C^) [1]. These diseases include scrapie in sheep, bovine spongiform encephalopathy in cattle, chronic wasting disease in cervids, and sporadic, genetic, or acquired human prion diseases [2]. The most common human prion disease, the sporadic form of Creutzfeldt–Jakob disease (sCJD), is clinically characterized by rapidly progressive dementia, neurologic dysfunction including myoclonic involuntary movements, and, finally, a terminal state of severe cognitive impairment leading to death within months from the onset of clinical symptoms [3]. Various clinical phenotypes can be observed, e.g., patients with ataxia opposed to dementia as the initial symptom or patients with anopsia as a prominent clinical feature. This marked clinical heterogeneity observed in sCJD is reflected by the presence of diverse sCJD types [4] and is not yet fully understood.

It is known that various isolates, or strains, of prions may be propagated within genetically identical hosts, leading to distinct clinical and pathological features [5,6]. The apparent diversity of human prion diseases may, at least in part, be attributed to the presence of distinct prion strains in affected individuals. In agreement with this, distinct clinical phenotypes of human prion diseases present with diverse deposition patterns of PrP^Sc^ in addition to disparate biochemical properties of PrP^Sc^ [7,8]. Since nucleic acids do not copurify with prion infectivity and prion strains do not seem to be encoded by differences in PrP primary structure, the hypothesis that PrP^Sc^ itself may encode strain-specific phenotypic properties within its tertiary or even quaternary structure has to be considered [9,10]. This assumption is supported by the presence of strain-specific N-terminal cleavage of PrP^Sc^ upon Western blot analysis following limited digestion with proteinase K [9].

Another important factor in phenotype variability is the host genotype of the gene encoding the prion protein *(PRNP)*. It is well established that the polymorphism at codon 129, encoding either methionine or valine in *PRNP,* influences the disease phenotype [8].

Thus, the classification of human prion diseases is based on the clinical presentation of the affected individual, *PRNP* status in concert with neuropathological findings, and the biochemical analysis of PrP^Sc^ [11–13]. Previous studies have focused on the assessment of the fragment sizes of unglycosylated PrP^Sc^ and the ratios of the three glycoforms of PrP^Sc^ as seen on Western blot following protease treatment [12,13]. Although there is some disparity in the exact number of mobilities of the unglycosylated PrP^Sc^ fragment, researchers agree that this fragment principally migrates between 19 and 21 kDa [12,13]. Since the resolution of one-dimensional gel electrophoresis is limited, it is likely that additional species of N-terminal fragments will be identified if more accurate techniques, such as two-dimensional immunoblot or mass spectrometry, are utilized. In fact, recent studies using two-dimensional gel electrophoresis of human sCJD brain homogenates strongly suggest that this is the case [14,15]. In order to simplify comparability of our study to published work, we decided to distinguish two principal mobilities of unglycosylated PrP^Sc^ i.e., 21 kDa (high) and 19 kDa (low), thereby accepting that both the high and the low PrP^Sc^ fragments show a certain degree of variation.

In agreement with the fact that the predominant PrP^Sc^ type may be indicative of the sCJD type, previous studies have demonstrated that the co-existence of more than one PrP^Sc^ type is a rare event [8]. In the light of these data, the finding that the co-existence of more than one PrP^Sc^ type within one individual occurs in 30% of all patients if multiple regions of the brain are investigated [16] came as a surprise. Because of the limited sample size in the latter study, the question of the true incidence of co-existence of more that one PrP^Sc^ type within one individual has remained unanswered.

It has been shown that the spatial analysis of histopathological changes often referred to as the lesion profile may provide a tool to discriminate various clinically diverse types of prion diseases [8,17]. A major drawback of this method, however, is the considerable inter-observer variability. Thus it has been virtually impossible to compare published lesion profiles [8,13].

Here, we assess the distribution of PrP^Sc^ types in nine central nervous system regions in a cohort of 50 patients with sCJD, using PrP^Sc^ profiling. Furthermore, we address the question of co-existence of more than one PrP^Sc^ type in sCJD-affected individuals.

## Methods

### Selection of Patients

Cases in this study were derived from an unselected series of patients with clinically, genetically, and neuropathologically proven sCJD (Tables 1 and 2). All tissue specimens originated from patients referred to the Swiss National Reference Centre for Prion Diseases, and the specimens were processed according to established guidelines regarding safety and ethics. Frozen brain tissue was stored at –80 °C and samples were taken from the following brain regions: frontal, parietal, occipital, and temporal cortex, and the putamen, thalamus, midbrain, medulla oblongata, and cerebellum.

**Table 1:**
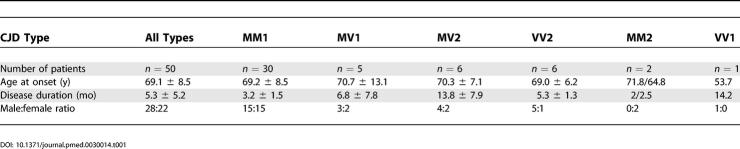
Demographic Characteristics and Classification of Patients with sCJD Included in the Study

**Table 2:**
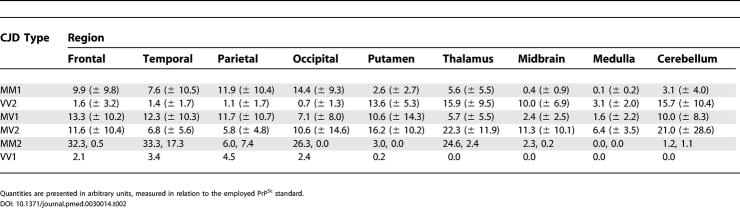
PrP^Sc^ Quantities in Defined Brain Areas

For positive control, neuropathologically proven sCJD cases (Codon 129 VV, PrP^Sc^ type 2 and Codon 129 MM, PrP^Sc^ type 1) were chosen. For negative control, a non-demented, age-matched autopsy case was utilized.

### Protein Analysis

Brain-tissue homogenates (10% w/v) were prepared in homogenization buffer (100 mM NaCl, 10 mM EDTA, 100 mM Tris-HCl, 0.5% NP40, 0.5% NaDOC [pH 6.9]) using a RiboLyser (Hybaid, Ashford, United Kingdom) and stored at –80 °C until use. Samples, containing 25 μg of protein, were digested with Proteinase K (PK-recombinant PCR-grade solution with a specific activity of 41.3 U/mg, measured by a hemoglobin-activity test [Roche, Basel, Switzerland]) for 30 min at 37 °C with a concentration of 0.03 U (40 μg/ml) per sample. Proteinase K was stored at –80 °C in storage buffer (50% glycerol, 10 mM Tris-HCl [pH 7.5], 2.9 mg/ml CaCl_2_). Proteins were separated on a 12% SDS-PAGE (10 × 10.5 cm, Hoefer, San Francisco, California, United States) and then transferred (1.5 h at 250 mA) to a nitrocellulose membrane (Protran, Schleicher & Schuell, Dassel, Germany) using a wet-blotting system (Bio-Rad, Hercules, California, United States). Membranes were incubated overnight with monoclonal antibody 3F4 [18] (Signet, Dedham, Massachusetts, United States) at a dilution of 1:2,000. After washing, HRP-conjugated rabbit-anti-mouse-IgG-γ (Zymed, San Francisco, California, United States) served as the secondary antibody at a dilution of 1:20,000. The signal was visualized by enhanced chemiluminescence using a VersaDoc 5000 imaging station (Bio-Rad). Cases were typed according to the size of the protease-resistant unglycosylated PrP fragment [12,13], and relative quantification of the signal was performed employing Quantity One software (Bio-Rad).

To quantify PrP^Sc^, serial dilutions of a standard sCJD brain homogenate (33, 16.5, and 8.25 μg of protein) were used on each Western blot to generate a standard curve. Quantification was performed only if the square of the correlation coefficient of the standard curve was above 0.93 (*r^2^* > 0.93).

Selected cases were separated by SDS-urea-PAGE. For this, we adapted a published protocol [19]. Briefly, the stacking gel was composed of 11.1% of a 60% acrylamid/0.8% bis-acrylamid mixture, 27.7% Tris-HCl 0.6M (pH 6.8), 1.3% SDS, 1.6% APS, 0.2% Temed (*N,N,N,N,*-tetra-methyl-ethylenediamine [Bio-Rad]), and 8 M urea, and the resolving gel was comprised of 44% of a 60% acrylamid/0.8% bis-acrylamid mixture, 53.9% Tris-HCl 1.875 M (pH 8.8), 1.4% SDS, 0.6% APS, 0.1% Temed (*N,N,N,N*,-tetra-methyl-ethylenediamine [Bio-Rad]), and 8 M urea.

### *PRNP* Analysis

Genomic DNA was extracted from the buffy-coat fraction of peripheral blood or from brain tissue. The complete *PRNP* open reading frame was analyzed using established methods [20].

### Neuropathology

Tissue from selected patients (*n* = 32) was fixed in 10% formalin. Tissue blocks from frontal, parietal, occipital, and temporal cortex, and the putamen, thalamus, midbrain, medulla oblongata, and cerebellum, localized adjacent to the specimens used for biochemical analysis, were selected, decontaminated for 1 h with 98% formic acid and embedded in paraffin. Sections (3 μm) were subjected to conventional staining and to immunostaining for glial fibrillary acidic protein (Dako), and for PrP (3F4), upon hydrolytic autoclaving according to published protocols [20].

Spongiosis was evaluated on a 0–4 scale (not detectable, mild, moderate, severe, and status spongiosus). Gliosis and PrP^Sc^ content were scored on a 0–3 scale (not detectable, mild, moderate, severe). PrP^Sc^ accumulation patterns were described as synaptic, perivacuolar, patchy, or plaque-like. Averaging of the three scores resulted in the value that was employed in order to obtain the lesion profile for individual patients [8]. Histological analysis was performed by two independent investigators blinded to clinical data, codon-129 status, and PrP biochemistry.

## Results

### Reliable Quantification of PrP^Sc^ Using Internal Standards

**Figure 1:**
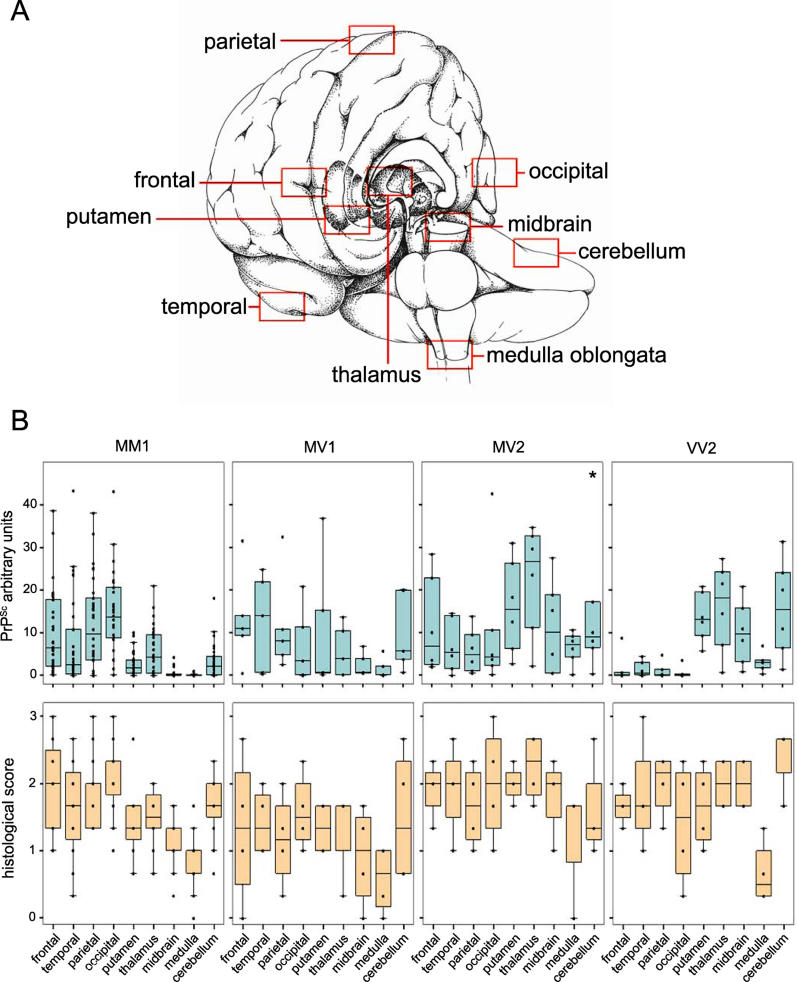
Comparison of PrP^Sc^ Profiles to Lesion Profiles (A) Schematic drawing indicating sampled areas which are used for the generation of PrP^Sc^ profiles (red boxes). (B) Biochemical PrP^Sc^ profiles are indicated in the upper panel, whereas histological lesion profiles are shown in the lower panel. Patients were grouped according to [12] in MM1, MV1, MV2, and VV2. Brain regions are shown on the *x*-axis. Values for PrP^Sc^ amounts are given in arbitrary units measured in relation to the PrP^Sc^ standard. Values for lesion profiles were obtained by averaging the scores for spongiosis (scored on a scale from 0–4), astrogliosis, and PrP immunoreactivity (both scored on a scale from 0–3). Black dots represent individual patients and black lines within boxes represent medians; boxes encompass 25th and 75th percentiles of distribution. One outlier (denoted by asterisk: MV2, cerebellum, PrP^Sc^ content = 84.1) has been omitted in the graphical representation.

Routinely, quantification of PrP^Sc^ is performed by comparing the signal intensities on Western blot analysis following limited proteinase K digestion. While this method is highly specific and objective, it does not allow for the comparison of samples run on separate gels owing to variations in the blotting process, antibody binding efficiencies, or differences in visualization reaction. In order to circumvent these problems, we developed a system whereby these variables are compensated for by quantifying the signal intensity of a serially diluted PrP^Sc^ standard on every Western blot. The resulting standard curve is then used to quantify PrP^Sc^ content in defined brain regions. The reliability and feasibility of this method was assessed by analyzing nine different brain areas on a cohort of 50 patients with sCJD (see Table 1; Figure 1). Standard curves were accepted only if the square of the correlation coefficient was above 0.93. Our PrP^Sc^ standard is available to the scientific community and should facilitate comparison of PrP^Sc^ quantities present in defined samples.

### Molecular Analysis of sCJD Using PrP^Sc^ Profiling

Nine defined brain areas of 50 subjects with sCJD were analyzed. They showed a mean age of 69.1 y (± 8.5) and a gender ratio of 28 males to 22 females. Patients were grouped according to both their genotype at codon 129 of the *PRNP* gene and to their predominant PrP^Sc^ type (Table 1), in line with the classification introduced by Parchi [8], and included MM1 (*n* = 30), MV1 (*n* = 5), MV2 (*n* = 6), VV2 (*n* = 6), MM2 (*n* = 2), and VV1 (*n* = 1).

Analysis of the relative amounts of PrP^Sc^ in various brain regions revealed distinct distribution patterns, which seem to be dependent on the type of PrP^Sc^ and on the host's codon-129 polymorphism. Cases with a slower-migrating unglycosylated band of PrP^Sc^ (∼21 kDa) and homozygosity for methionine on codon 129 (MM type 1) harbor significant amounts of PrP^Sc^ in cortical regions (frontal, parietal, temporal, and occipital) and the thalamus, smaller and variable amounts in the putamen and cerebellum, and very low or undetectable amounts in the midbrain and medulla oblongata (Figure 1). PrP^Sc^ quantities for this cohort are listed in Table 2.

In contrast, cases with a faster-migrating unglycosylated band of PrP^Sc^ (∼19 kDa) and homozygosity for valine on codon 129 (VV type 2) had only scant amounts of PrP^Sc^ in the cerebral cortex, but showed marked PrP^Sc^ deposition within the putamen, thalamus, the midbrain, the cerebellum and, to a lesser extent, in the medulla oblongata, although this was clearly detectable (Figure1). PrP^Sc^ quantities for this cohort are listed in Table 2.

Whereas the deposition patterns described above were relatively uniform in the respective groups, the picture is not so obvious for patients heterozygous for methionine or valine at codon 129. In this group, patients with a slower-migrating unglycosylated band of PrP^Sc^ (∼21 kDa, MV1) exhibited deposition patterns reminiscent of MM1 individuals. However, PrP^Sc^ deposition was more abundant in the temporal than in the occipital lobe, and deposits within the putamen showed greater variability (Figure 1).

Patients with a faster-migrating unglycosylated band of PrP^Sc^ (∼19 kDa, MV2) exhibited abundant PrP^Sc^ deposition in the putamen, thalamus, midbrain, and cerebellum, clearly detectable deposits in the medulla, and, in addition, variable deposits within the cerebral hemispheres (Figure 1). PrP^Sc^ quantities for these two cohorts and for patients either homozygous for methionine (MM2, *n* = 2) or valine (VV1, *n* = 1) are listed in Table 2.

Because this method possesses some similarities with lesion profiling, usually used to discriminate between sCJD types or between prion strains in rodents, we have termed this method “PrP^Sc^ profiling” [8,17].

### High Proportion of PrP^Sc^ Type Co-Existence in Patients Expressing MV at Codon 129

Studies on relatively small cohorts of patients with sCJD evidenced the co-occurrence of different PrP^Sc^ types within one individual [16]. We analyzed nine distinct brain regions of 50 patients with sCJD, resulting in a total of 450 analyzed specimens. In patients where routine SDS-PAGE analysis suggested possible co-occurrence of distinct PrP^Sc^ types, high-resolution SDS-urea-PAGE was performed (*n* = 13).

**Figure 2:**
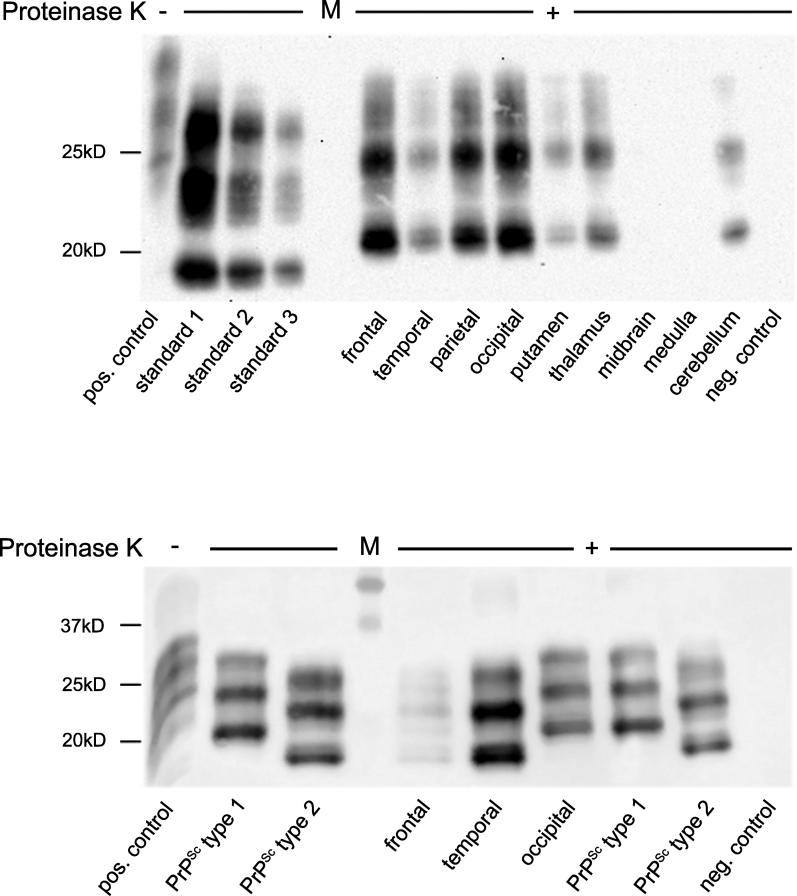
Western Blots Illustrating the Principle of PrP^Sc^ Profiling and the Co-Occurrence of Multiple PrP^Sc^ Types within One Patient with sCJD (Upper panel) The PrP^Sc^ content in various central nervous system regions (indicated below respective lanes) was quantified by plotting the signal intensity on a standard curve created by a serially diluted PrP^Sc^ standard (standard 1 to 3). Proteinase K digestion is indicated above lanes. (Lower panel) Presence of a faster-migrating unglycosylated band of PrP^Sc^ (∼19 kDa, PrP^Sc^ type 2, frontal and temporal cortex) and a slower-migrating unglycosylated band of PrP^Sc^ (∼21 kDa, PrP^Sc^ type 1, occipital cortex) within one patient with sCJD (codon 129 MM). Controls include PrP^Sc^ type 1 and 2 and a sample from a non prion-diseased individual. Proteinase K digestion is indicated above lanes.

A total of nine sCJD individuals showed non-ambiguous co-occurrence of distinct PrP^Sc^ types as evidenced by the presence of a faster- and a slower-migrating unglycosylated band of PrP^Sc^, either in different brain areas or within one brain region (Figure 2). In the remainder of the cases (*n* = 41), only a single PrP^Sc^ type could be detected in all nine different brain areas. In cases where more than one PrP^Sc^ type was observed, the topological distribution of distinct strain types revealed no obvious pattern (Table 3). However, the cerebral hemispheres appeared to be less affected than the thalamus, putamen, cerebellum, midbrain, and medulla oblongata. Whereas co-occurrence of both PrP^Sc^ types was found in only two cases within the frontal lobe, the midbrain was affected in three cases, the medulla in four cases, and the putamen, thalamus, and cerebellum each in five cases.

**Table 3:**
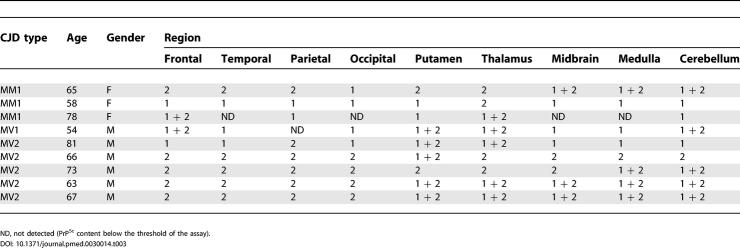
Different PrP^Sc^ Types in Defined Central Nervous System Regions of sCJD Patients

The most striking feature of patients harboring two PrP^Sc^ types was their association with the methionine/valine polymorphism at codon 129. Six out of 11 methionine/valine heterozygotes displayed both PrP^Sc^ types, whereas only three of 32 individuals homozygous for methionine produced more than one PrP^Sc^ type. In the groups of patients expressing valine/valine on codon 129, we were not able to detect more than one PrP^Sc^ type.

### Neuropathological Analysis

The analysis of the neuropathological lesion pattern was performed according to published protocols [8,13] with the purpose to: (i) evaluate whether immunohistochemical PrP staining and histological lesions (gliosis, spongiosis) correlate with the absolute amount of biochemically assessed PrP^Sc^, and (ii) compare whether lesion profiles generated by histological assessment parallel biochemically defined PrP^Sc^ profiles. In order to facilitate these analyses, patients with sCJD were subdivided into following groups: MM1, MV1, MV2, VV2 [12]. Patients presenting with multiple PrP^Sc^ types were allocated according to the dominating PrP^Sc^ type.

The MM1 group (*n* = 20) showed marked involvement of the frontal and temporal cerebral cortex and slightly fewer lesions in the cerebellum, whereas the putamen, thalamus, midbrain, and medulla oblongata showed relatively modest pathological alterations (see Figure 1).

In the MV1 cohort (*n* = 4), a similar lesion profile could be observed (Figure 1). The observation that this group showed a greater variability might in part be attributable to the smaller sample size.

MV2-predominant patients (*n* = 4) exhibited a consistently high degree of neuropathological lesions with relative sparing of the occipital cortex and the cerebellum.

In the VV2 group (*n* = 4), pathological alterations were most pronounced in the cerebellum and in the cerebral cortex, as well as in the thalamus and midbrain. The medulla oblongata did not show a prominent involvement (Table S1).

The correlation between histological lesions as assessed by lesion profiling and the presence of PrP^Sc^ evaluated by PrP^Sc^ profiling was not striking. Although brain regions with abundant PrP^Sc^ tend to show severe histopathological lesions, the reverse does not always seem to be the case. Brain regions with massive histopathological lesions (i.e., cortical regions in VV2 patients) show only scarce depositions of PrP^Sc^.

The comparison of lesion profiles to PrP^Sc^ profiles demonstrates that the patterns obtained within the above-mentioned groups are only roughly concurrent. Whereas PrP^Sc^ profiles show marked differences between investigated regions, lesion profiles tend to show a more homogeneous picture with fewer marked regional differences.

**Figure 3:**
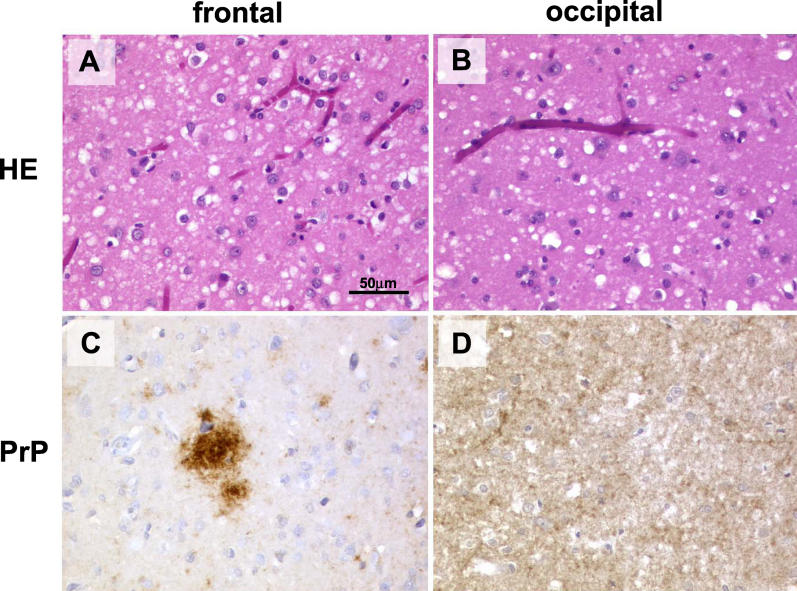
Histological Analysis of Central-Nervous-System Sections in One Patient with sCJD with Co-Occurrence of Multiple PrP^Sc^ Types H&E-stained (A and B) and PrP-immunostained (C and D) cortical areas showing pronounced spongiosis and deposition of PrP^Sc^ in a plaque-like (C; PrP^Sc^ type 2, see Figure 2, frontal area on the lower panel) and synaptic (D; PrP^Sc^ type 1, see Figure 2, occipital area on the lower panel) pattern.

In cases in which multiple PrP^Sc^ types could be observed, we investigated whether the predominant deposition pattern of PrP^Sc^ was associated with a particular biochemical type of PrP^Sc^. Areas with a predominance of PrP^Sc^ type 1 (∼21-kDa unglycosylated band of PrP^Sc^) displayed diffuse, synaptic PrP immunoreactivity, whereas areas with a PrP^Sc^ type 2 (∼19-kDa unglycosylated band of PrP^Sc^), showed mainly perivacuolar or plaque-like deposits of PrP (Figure 3).

## Discussion

The precise classification of sCJD by molecular, histopathological, and clinical parameters is important for understanding the underlying pathogenesis, unraveling possible etiological causes, and devising efficient therapeutic protocols. Several classification systems for human prion diseases have been proposed in the past [8,13]. Although the exact number of molecular subtypes is still a matter of debate, there is a consensus that these may be differentiated on the basis of clinical data, histopathological analysis, codon-129 status of *PRNP,* and biochemical analysis of PrP^Sc^.

The approach described here represents a new method for the discrimination of molecular subtypes of human prion diseases. By quantifying PrP^Sc^ in nine brain regions of patients with sCJD, we were able to determine the PrP^Sc^ profile of 50 patients with sCJD. Because individual measurements are calibrated to internal standards, this method allows, for the first time, for absolute quantification of PrP^Sc^ in prion-diseased individuals, and therefore enables the direct comparison of individual PrP^Sc^ profiles, excluding inter-observer and methodological variations. Detailed analysis of different molecular subtypes of sCJD confirmed the feasibility of this method and showed subtype-specific differences in the regional distribution of PrP^Sc^. Besides advancing the molecular analysis of sCJD, these data demonstrate that if confirmation of sCJD by brain biopsy is indispensable, it should only be carried out taking into account the sCJD subtype. In patients belonging to the MM1 groups [8], high amounts of PrP^Sc^ are present in all cortical areas and would favor a cortical biopsy site. In patients belonging to the VV2 group [8], minimal amounts of PrP^Sc^ in the cerebral cortex would designate the cerebellum or the thalamus as sites for successful confirmation of the disease by biopsy.

An earlier study that attempted to assess the distribution of PrP^Sc^ within the central nervous system was performed only on a small patient cohort and did not employ internal PrP^Sc^ standards–thus impeding the direct comparison of analyses [16]. In other studies, lesion profiles were based on histological scores [8,13,17]. Although this histological, score-based method has certain advantages and can be carried out on fixed tissue, we believe that PrP^Sc^ profiling will be a valuable tool for prion research. Since both our work and other studies show that there is no strict correlation between histopathological alterations and the presence of PrP^Sc^, one could assume that PrP^Sc^ profiling is superior since it measures PrP^Sc^, which is believed to constitute an essential component of prion infectivity [4,16].

Even though experiments in rodents performed some years ago demonstrated the phenomenon of multiple PrP^Sc^ types within one animal [21], incongruity regarding the frequency of co-occurrence of different PrP^Sc^ types in sCJD patients still endures. Initial studies suggested that this is a relatively rare event, occurring in less than 5% of patients [8]. This low incidence may, at least partially, be attributed to the fact that these analyses are routinely performed on a limited range of distinct brain regions per patient [8,13]. The fact that region-specific presence of distinct PrP^Sc^ types may occur in sCJD was highlighted by a publication investigating ten defined regions within the central nervous system in 14 patients with sCJD [16]. The authors found more than one PrP^Sc^ type in five individuals and hypothesized that the co-occurrence of more than one PrP^Sc^ type could be the rule, rather than the exception, if the entire central nervous system was investigated. In our analysis of nine distinct central-nervous-system regions in 50 patients with sCJD using a standardized protocol [22], we detected more than one PrP^Sc^ type in nine individuals. The observation that most patients harboring two PrP^Sc^ types are codon-129 methionine/valine heterozygotes stresses the significance of this polymorphism in the replicative cycle of PrP^Sc^.

Analysis of the spatial distribution of PrP^Sc^ types revealed that the putamen, thalamus, medulla oblongata, and cerebellum are prone to accommodate multiple PrP^Sc^ types simultaneously, whereas this was rarely observed in the cerebral cortex. The fact that central nervous system regions which simultaneously harbor two PrP^Sc^ types are complex structures containing diverse subsets of neuronal cells indicates that susceptibility of target cells may, at least partially, determine the type of PrP^Sc^ that is produced. This finding is compatible with the target-cell hypothesis of prion-strain diversity [23,24].

In conclusion, the present study has introduced a new method for the characterization of human prion diseases. The approach described here will aid in understanding the molecular diversity of this disease entity. Furthermore, these data imply that the subsets of cells present in specific brain areas in concert with the polymorphism on codon 129 of *PRNP* may have a fundamental role in determining which PrP^Sc^ type is preferentially replicated.

## Supporting Information

Table S1 Histological Assessment of the VV2 Subgroup of Patients Cases 1 to 4: spongiosis is scored on a scale from 0–4; astrogliosis and PrP immunoreactivity are scored on a scale from 0–3.
(41 KB DOC).

**Patient Summary**

**Background**

Prions cause neurodegenerative diseases in humans and in animals like cows, sheep, or deer. In most cases, humans get sick sporadically, when, for unknown reasons, normal proteins in the brain (called PrP^c^) change to harmful prions (called PrP^Sc^). The PrP^Sc^ proteins then cause severe degeneration of the brain, which causes death within a matter of months. The most common human prion disease is sporadic Creutzfeld-Jacob Disease, or sCJD. The disease is rare (it affects roughly one in a million people worldwide), and there is no cure or therapy. Scientists still understand little about why and how, in some cases, normal proteins change to the harmful prions that destroy the brain. Although all patients with prion disease die, there are differences between individual cases. Neurological symptoms, particularly early in the disease, are variable. Scientists have also found that there are different versions of the gene that codes for the normal PrP^c^ protein. Moreover, in some patients all of the prions found in the brain after autopsy are alike, whereas in other patients there seem to be several types of prions. In addition, brain autopsies have shown differences in the amount of prions and in the extent of damage present in different parts of the brain.

**Why Was This Study Done?**

The hope is that understanding the differences between individual cases might eventually provide some insight into the causes of the disease and suggest ways to treat it. To catalogue and examine those differences, however, it is important to come up with standard assays that allow meaningful comparisons. The authors of this study wanted to develop such assays.

**What Did the Researchers Do and Find?**

They developed an assay, which they called PrP^Sc^ profiling. The new assay measures the amount of prions in different parts of the brain in a way that makes it easy to compare autopsy samples from different patients analyzed at different times and by different people. They then used this assay to study autopsy samples from 50 patients for whom they knew the disease symptoms and the genetic make-up. For each patient sample, they examined nine different brain regions for the amount of prions present. They also checked whether all of the prions were alike or whether there were different types of prions. They found that there were distinct prion-distribution patterns in different sCJD subtypes. In about 20% of the patients, they found more than one prion type. Many of these patients had a specific genetic make-up, and the mixture of prion types was mostly seen in a few specific brain regions.

**What Do These Findings Mean?**

PrP^Sc^ profiling should prove to be useful for the classification of human prion diseases and should allow scientists worldwide to compare their samples. The specific results in the 50 patient samples will encourage other researchers to look for correlations between disease subtypes and prion-distribution patterns, and to further explore the link between genetic make-up, specific brain region, and the existence of more than one prion type. The hope is that the combined data will help scientists to understand the disease and to come up with ways to prevent or treat it.

**Where Can I Get More Information Online?**

The following websites provide information on prion diseases.

The National Creutzfeldt–Jakob Disease Surveillance Unit in Edinburgh, United Kingdom:

http://www.cjd.ed.ac.uk

The European and Allied Countries Collaborative Study Group of CJD:

http://www.eurocjd.ed.ac.uk

US National Institute of Mental Health pages on prion diseases:

http://www.ninds.nih.gov/disorders/tse/tse.htm

US Centers of Disease Control and Prevention pages on prion diseases:

http://www.cdc.gov/ncidod/dvrd/prions

Helpguide pages on Creutzfeld-Jacob disease:

http://www.helpguide.org/elder/creutzfeldt_jakob.htm

Wikipedia pages on prions:

http://en.wikipedia.org/wiki/prions

## Abbreviations

sCJD: sporadic Creutzfeldt–Jakob disease
