# Supplementary material for: Analysis of Prion Strains by PrPSc Profiling in Sporadic Creutzfeldt–Jakob Disease
Source: PLoS Med. 2005 Dec 20;3(2):e14. doi: 10.1371/journal.pmed.0030014 (PMC1316067; doi:10.1371/journal.pmed.0030014)
Supplement: Supplementary file 1 [file pmed.0030014.st001.doc]

Supplemental material

Table 4: Histological assessment of the VV2 subgroup of patients (cases 1 to 4, spongiosis is scored on a scale from 0-4, astrogliosis and PrP immunoreactivity are scored on a scale from 0-3).

|  | case | region (score) | | | | | | | | |
| --- | --- | --- | --- | --- | --- | --- | --- | --- | --- | --- |
|  |  | frontal | temporal | parietal | occipital | thalamus | putamen | midbrain | medulla | cerebellum |
| spongiosis | 1 | 1 | 1 | 2 | 0 | 2 | 2 | 2 | 1 | 2 |
|  | 2 | 2 | 1 | 2 | 2 | 1 | 2 | 2 | 1 | 2 |
|  | 3 | 2 | 3 | 3 | 3 | 2 | 3 | 2 | 2 | 1 |
|  | 4 | 1 | 0 | 1 | 0 | 2 | 2 | 2 | 0 | 2 |
|  |  |  |  |  |  |  |  |  |  |  |
| gliosis | 1 | 2 | 2 | 3 | 2 | 2 | 2 | 3 | 1 | 3 |
|  | 2 | 2 | 2 | 2 | 3 | 2 | 3 | 2 | 0 | 3 |
|  | 3 | 2 | 3 | 2 | 2 | 2 | 2 | 1 | 0 | 2 |
|  | 4 | 1 | 1 | 2 | 0 | 2 | 1 | 2 | 1 | 3 |
|  |  |  |  |  |  |  |  |  |  |  |
| PrP | 1 | 2 | 2 | 2 | 1 | 0 | 1 | 2 | 0 | 3 |
|  | 2 | 1 | 2 | 2 | 1 | 0 | 2 | 1 | 0 | 3 |
|  | 3 | 2 | 3 | 2 | 2 | 3 | 2 | 2 | 2 | 2 |
|  | 4 | 2 | 2 | 1 | 1 | 2 | 2 | 3 | 0 | 3 |
